# Molecular characterization, purification, and antioxidant activity of recombinant superoxide dismutase from the Pacific abalone *Haliotis discus hannai* Ino

**DOI:** 10.1007/s11274-020-02892-5

**Published:** 2020-07-14

**Authors:** Kun Qiao, Chunhua Fang, Bei Chen, Zhiyu Liu, Nan Pan, Hui Peng, Hua Hao, Min Xu, Jingna Wu, Shuji Liu

**Affiliations:** 1 Fisheries Research Institute of Fujian, Key Laboratory of Cultivation and High-Value Utilization of Marine Organisms in Fujian Province, Xiamen, 361013 China; 2 The Public Service Platform for Industrialization Development Technology of Marine Biological Medicine and Product of State Oceanic Administration, Fuzhou, 350117 China; 3 College of Ocean and Earth Sciences, Xiamen University, Xiamen, 361013 China

**Keywords:** Antioxidant, *Haliotis discus hannai*, *Pichia pastoris*, Superoxide dismutase

## Abstract

**Abstract:**

Superoxide dismutase (SOD) is an acidic metalloenzyme that scavenges free radicals produced by endogenous and exogenous substances. In the present study, the tissue distribution of the superoxide dismutase HdhCu/Zn-SOD was investigated in *Haliotis discus hannai* Ino. The expression profile after lipopolysaccharide (LPS) challenge was determined using quantitative real-time polymerase chain reaction (qPCR). To study the antioxidant activity of a recombinant HdhCu/Zn-SOD protein, the HdhCu/Zn-SOD gene was cloned into the pPIC9K vector and transformed into the *Pichia pastoris* GS115 strain by electroporation. After induction by methanol, the recombinant product was purified using immobilized metal affinity chromatography and confirmed using mass spectrometry. The optimal expression conditions were determined to be incubation with 0.5% methanol at pH 6.0, resulting in a stable expressed product with the molecular weight of approximately 17 kDa and 21 kDa. The enzymatic activity of HdhCu/Zn-SOD consistently increased with increasing Cu^2+^ concentrations and showed good thermal stability. Recombinant HdhCu/Zn-SOD showed a strong ability to scavenge superoxide anions and hydroxyl radicals and protected L929 cells against the toxicity caused by H_2_O_2_ through its in vitro antioxidant activity. The heterologous expression of HdhCu/Zn-SOD in *P. pastoris* and the antioxidant activity of this enzyme are reported for the first time.

**Graphic abstract:**

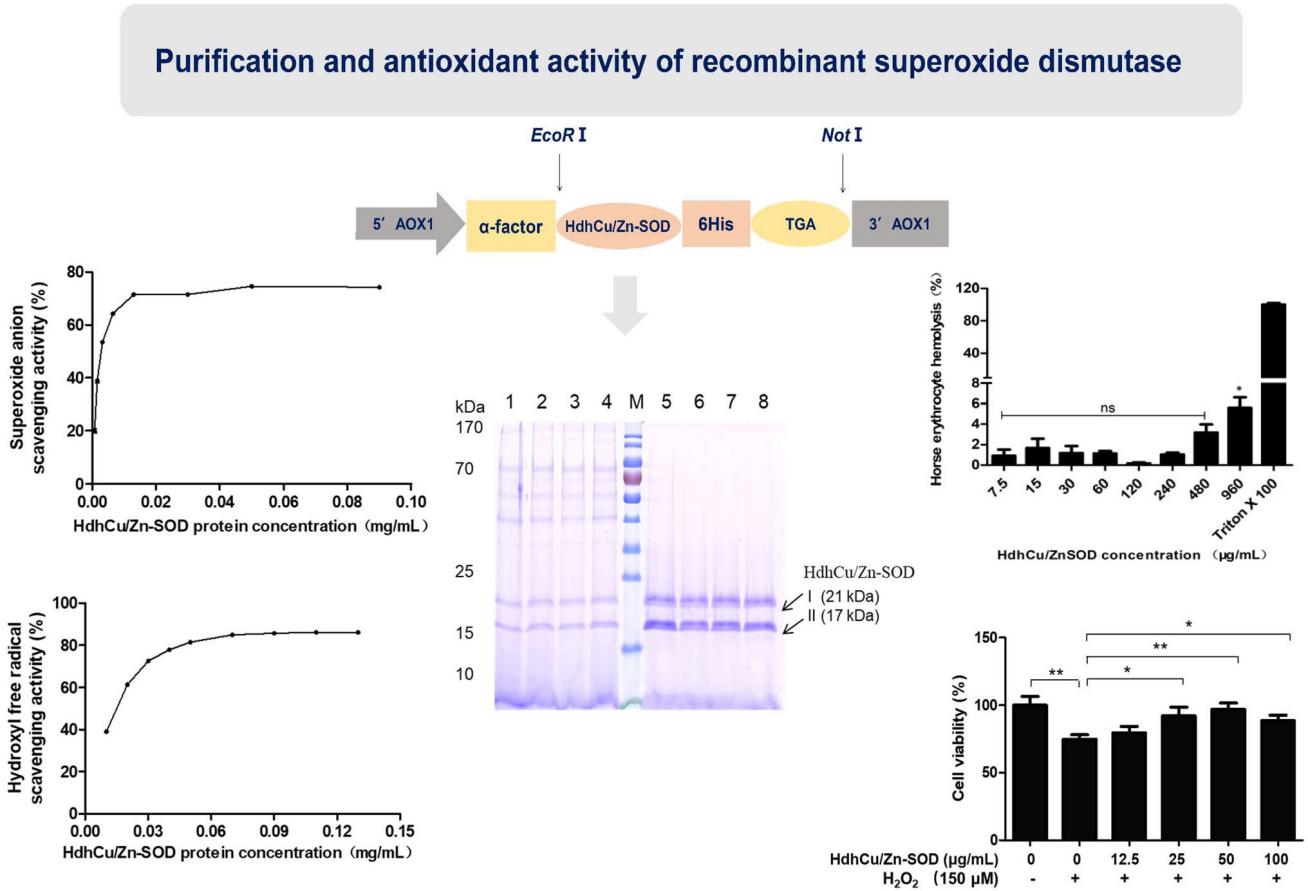

**Electronic supplementary material:**

The online version of this article (10.1007/s11274-020-02892-5) contains supplementary material, which is available to authorized users.

## Introduction

Marine organisms exposed to a complex aquatic environment show increased antioxidant responses. Reactive oxygen species (ROS) are oxygen free radicals that are generated during aerobic cellular metabolism, such as superoxide anions (O_2_-), hydroxyl radicals (.OH), single oxygen (.O_2_), and hydrogen peroxide (H_2_O_2_) (Genestra 2007). In organisms, ROS remain in dynamic equilibrium under normal physiological conditions (Finkel and Holbrook 2000). Under inflammation, the oxidase enzyme is activated in host organisms, leading to the production of ROS that kill invading pathogens (known as respiratory burst Hsieh et al. 2008;). However, many types of environmental stimuli, including ultraviolet light, environmental toxins, and hyperthermia, generate high levels of ROS, resulting in cellular oxidative damage. ROS can degrade unsaturated fatty acids on the cell membrane and destroy the membrane structure (Sargis and Subbaiah 2006), leading to the cleavage of polypeptide chains (Stadtman and Levine 2003) and the formation of single-strand breaks in nuclear DNA (Melidou et al. 2005). To avoid cellular oxidative damage and prevent disease and death caused by oxidative stress, marine organisms develop an effective defense system to maintain the balance of ROS. The antioxidant system of mollusks includes enzymatic antioxidants such as superoxide dismutase (SOD, EC1.15.1.1), catalase, thioredoxin peroxidase, glutathione peroxidase, and glutathione-S-transferase and nonenzymatic antioxidants such as selenium-binding protein, metallothionein, thioredoxin, ferritin, and transferrin.

Superoxide dismutase is an acidic metalloenzyme that is ubiquitous in animals, plants, and microorganisms. In vivo, its role is to scavenge free radicals produced by endogenous and exogenous substances, inhibit lipid peroxidation, and delay senescence to protect the organism (Che et al. 2016; Fukai and Ushio-Fukai, 2011). SOD can also improve phagocytosis and viral resistance in cells and is therefore often used as a potential marker for the assessment of environmental stress and water pollution (Achuba 2002; Penicaud et al. 2017; Ren et al. 2017). SODs are mainly divided into three types depending on their metal co-factor: iron SOD (Fe-SOD), manganese SOD (Mn-SOD) and copper and zinc SOD (Cu/Zn-SOD) (Smith et al. 2003; Wu et al. 2008). Cu/Zn-SOD can be further divided into extracellular Cu/Zn-SODs, with an N-terminal signal sequence, and intracellular Cu/Zn-SODs, without an N-terminal signal sequence (Lin et al. 2008). SODs can be divided into two main families based on their structure, one of which consists of Cu/Zn-SODs, while the other family comprises Fe-SODs and Mn-SODs (Lin et al. 2008). There are more available data and in-depth mechanistic studies on Cu/Zn-SODs than the other types. Researchers have also discovered rare SOD types such as a nickel-containing SOD (Ni-SOD) and an iron and zinc-containing SOD that is similar to Fe-SOD (Fe/Zn-SOD) in *Streptomyces* (Hong-Duk et al. 1996; Kim et al. 1996).

The Pacific abalone (*Haliotis discus hannai Ino*) is an economically important species in mariculture in China. Recently, the effects of water quality on mariculture in oceans and self-degradation of cultivated abalones have reduced disease resistance in artificially cultivated abalones. The antioxidant defense system plays an important role in defense and protection by ensuring the synthesis of protective ROS and preventing damage to the body. As the first-line enzymatic defense against superoxide, SOD scavenges free radical species produced from endogenous and exogenous substances. However, few studies have evaluated the detailed structure and protein function of antioxidant enzymes in marine invertebrates. These studies mainly involve cloning of antioxidant genes, genetic structure analyses, phylogenetic analyses, differential expression analyses, and in vitro prokaryotic expression of recombinant proteins, whereas there are fewer studies on functional validation at the protein level (Bao et al. 2008, 2009a, b; Han et al. 2016; Kim et al. 2011; Zheng et al. 2015). In order to gain insight into the innate immune defense mechanisms of abalones, we studied the expression characteristics of the SOD gene, which is the first line of defense against ROS. In this study, the tissue distribution of HdhCu/Zn-SOD was investigated in *H. discus hannai* Ino. To evaluate whether the HdhCu/Zn-SOD gene responds to immune-stimulant challenges, the expression profile after lipopolysaccharide (LPS) challenge was determined by quantitative real-time polymerase chain reaction (qPCR). Furthermore, we constructed a eukaryotic expression vector, pPIC9K-HdhCu/Zn-SOD, and induced the expression of this recombinant protein in the *Pichia pastoris* GS115 strain. The recombinant protein was purified by affinity chromatography, followed by antioxidant and cellular safety evaluation of the purified product. Study on the protective role of this recombinant protein against hydrogen peroxide-induced cell damage will develop its application in cosmetics, medicine, and other products as an additive.

## Materials and methods

### Animals and tissue collection

Pacific abalones (*H. discus hannai*) were purchased from the Dongshan abalone farm in Fujian Province and acclimatized in a laboratory environment for 7 days before the experiments were carried out. The animals were reared in 80 L polyvinyl chloride tanks containing 40 L of natural seawater, which was purified through a biofilter and renewed daily to ensure water quality. The animals were fed with the marine alga *Gracilaria tenuistipitata* during the acclimation and experimental period.

The abalones were divided into saline and LPS groups. LPS from *E. coli* (L2880, Sigma, USA) was dissolved in saline to 1 mg/mL for animal challenge. The abalones were injected with 50 µL of LPS or an equal volume of sterile saline via the front of the foot. The injection method was based on a previously published paper (Huang et al. 2010; Ren et al. 2009). Normal Pacific abalones were used as controls. Abalone hemocyte, gill, hepatopancreas, gonad, mantle, digestive gland, shell muscle, and epipodium samples were collected at 0, 4, 8, 12, 24, 48, and 96 h after injection. Samples from five abalones were collected from each group as parallel samples. The samples were rapidly placed in liquid nitrogen and stored at − 80 °C.

### Real-time PCR quantitation of HdhCu/Zn-SOD expression in Pacific abalones

The Trizol reagent was used to extract total RNA from the collected samples as previously described (Qiao et al. 2016; Liu et al. 2010). cDNA synthesis was carried out using the PrimeScript™ RT reagent kit (Perfect Real Time) (TaKaRa), following the manufacturer’s instructions. The HdhCu/Zn-SOD sequence was obtained from a transcriptome library in our previous study (GenBank accession no. KX302627). The real-time PCR system had a volume of 20 µL and included 10 ng of total RNA, 10 pmol of the specific primers HdhCu/Zn-SOD QS2 (5′-CAGTTCGGGGACAACACCAA-3′) and HdhCu/Zn-SOD QA2 (5′-TGTTTGCTACTCCTGATGCGT-3′), and 10 µL of FastStart Universal SYBR Green Master (Rox) (Roche). The reaction conditions were as follows: preincubation at 95 °C for 600 s, followed by 45 cycles (denaturation, 95 °C for 10 s; annealing, 60 °C for 10 s; and extension at 72 °C for 10 s). The β-actin (GenBank accession no. MN123625, β-actin QS2: 5′-TCTGCTACATCGCCCTTGAC-3′; β-actin QA2: 5′-AGGGACTCTGGACAACGGAA-3′) and Ribosomal protein L7 primers (GenBank accession no. KP698945, 7RPL-FW: 5′- CAAGCTGAACACTCCAAACG-3′; 7RPL-RV: 5′-TCCACAGCACTGATGTTTCC-3′) were designed to amplify the internal reference gene (Lee et al. 2016). qPCR data were calculated using the 2^−∆∆CT^ method. The melt-curve analysis was performed to analyze the specificity of the PCR products.

### Construction of the pPIC9K-HdhCu/Zn-SOD vector

The upstream primer F1: GGGGAATTCTCTATCAAAGCAGTTTGTG and the downstream primer R1: ATGCGGCCGCTCAATGGTGATGGTGATGATGCTTGG TGATGCCGATCA, which are specific to the Pacific abalone HdhCu/Zn-SOD gene, were designed based on the multiple cloning sites of the pPIC9K vector. The underlined sections are the endonuclease sites for *EcoR*I and *Not*I, respectively. A histidine tag was included before the stop codon; the boxed region is the 6 × His-tag. Protein expression and purification followed as previously described (Peng et al. 2012).

The HdhCu/Zn-SOD sequence was obtained from a transcriptome library in our previous study (GenBank accession no. KX302627). The Pacific abalone cDNA sequence was used as a template, and HdhCu/Zn-SOD S1 (5′-GGATTTATTCTCTTTGATAGACAGT-3′) and HdhCu/Zn-SOD A1 (5′-GACACCTCGGCAACCATTAC-3′) were used as upstream and downstream primers, respectively, to amplify the target gene sequence. Thereafter, the sequence was ligated to pMD18-T to obtain a recombinant-positive plasmid containing the HdhCu/Zn-SOD target gene. This positive plasmid was used as an amplification template, and HdhCu/Zn-SOD F1 and HdhCu/Zn-SOD R1 were used as upstream and downstream primers, respectively, to amplify the target gene sequence using TransStart FastPfu high-fidelity DNA polymerase (TransGen Biotech Co., Ltd.). The recovered Pacific abalone HdhCu/Zn-SOD target gene fragment (20 µg) was double digested with the *EcoR*I and *Not*I restriction endonucleases (TaKaRa Bio. Inc.). The pPIC9K vector was simultaneously subjected to double digestion with EcoRI and NotI. A DNA Ligation Kit (TaKaRa Bio. Inc.) was used to ligate pPIC9K and the HdhCu/Zn-SOD gene fragment at 16 °C. The ligation product was transformed into competent *E. coli* DH5α cells, which were then spread on an LB agar plate containing ampicillin and incubated at 37 °C. Positive clones were identified using PCR and submitted to Sangon Biotech (Shanghai) Co., Ltd. for DNA sequencing.

### Transformation of *P. pastoris* and PCR analysis of *P. pastoris* transformants

Bacterial strains containing the correct recombinant expression vector were streaked on a plate, and a single clone was selected for shaking incubation. After the plasmid was obtained, the *Sac*I restriction endonuclease (TaKaRa Bio. Inc.) was used for linearization. The nucleic acid precipitant was used to recover the linearized pPIC9K- HdhCu/Zn-SOD, which was transformed into competent *P. pastoris* GS115 cells by electroporation. The transformed cells were plated on minimal dextrose (MD) plates and cultured at 28 °C for 2 days. Single clones were selected for PCR amplification to verify whether the target gene fragment was inserted into the yeast genome using 5′AOX1 (5′-GACTGGTTCCAATTGACAAGC -3′) and 3′AOX1 (5′- GCAAATGGCATTCTGACATCC -3′) as primers.

### Eukaryotic expression of the pPIC9K-HdhCu/Zn-SOD vector and purification

A single colony on the MD plate was subcultured onto a yeast extract peptone dextrose medium (YPD) plate, which was then inverted for culture at 28 °C. A single colony was grown in 10 mL of buffered glycerol-complex medium (BMGY, 1% yeast extract, 2% tryptone, 1.34% yeast nitrogen base (YNB), 1% glycerol, 4 × 10^–5^% biotin, and 100 mM potassium phosphate buffer, pH 6.0) and cultured at 28 °C and 230 rpm until an OD_600_ of 2.0–6.0 was obtained. The culture was centrifuged at 2000 g for 5 min, and the supernatant was discarded. An equal volume of buffered methanol-complex medium (BMMY, 1% yeast extract, 2% tryptone, 1.34% YNB, 4 × 10^–5^% biotin, 0.5% methanol, and 100 mM potassium phosphate buffer, pH 6.0) was used to resuspend the cells, and the cells were divided. The pH was adjusted to 4, 5, 6, 7, or 8, and the yeast were incubated at 28 °C and 230 rpm for 24 h to induce expression. Different methanol concentrations (0.5%, 1.0%, 2.0%, 3.0%) were added. Pre-induction samples of the yeast cultures (1 mL) were collected at 0 h, 6 h, 12 h, 24 h, 48 h, 72 h, and 96 h. The samples were centrifuged at 12,000 g for 10 min at 4 °C, and 900 µL of supernatant was collected. Then, 100 µL of trichloroacetic acid was added to the supernatant for protein concentration. Sodium dodecyl sulphate–polyacrylamide gel electrophoresis (SDS-PAGE) electrophoresis was used to analyze the expression level of the target protein. The remaining supernatant of the expression culture was dialyzed three times in dialysis buffer. The solution was centrifuged, and the supernatant was filtered through a 0.45 μm filter membrane before purification.

Three to five column volumes of MilliQ water were used to wash the prepacked column (HisTrapTM FF Crude 5 mL), after which 3–5 column volumes of equilibration buffer (20 mM phosphate buffer + 50 mM NaCl + 10 mM imidazole, pH 8.5) were used to equilibrate the column. The filtered supernatant was loaded onto the column at 2 mL/min, and the eluate was collected. Next, 3–5 column volumes of equilibration buffer were used to wash away unbound protein from the column, after which elution buffer (20 mM phosphate buffer + 50 mM NaCl + 1 M imidazole, pH 8.5) was used to elute the protein. The elution peaks of the protein were collected, and small amounts were validated using SDS-PAGE electrophoresis.

### Enzyme activity assay

Various concentrations of CuSO_4_ (0, 0.2%, 0.5%, and 1%) were added to the induction medium, followed by culture for 24 h. The enzyme activity of the purified HdhCu/Zn-SOD protein was measured by the SOD assay kit (Nanjing Jiancheng Institute of Biological Engineering, China) according to manufacturer’s instructions, based on its ability to inhibit the oxidation of hydroxylamine by the xanthine—xanthine oxidase system (Ōyanagui, 1984). We assessed the effect of temperature on SOD enzyme activity. The enzyme samples were incubated for 10 min at 30 °C, 40 °C, 50 °C, 60 °C, 70 °C and 80 °C in 1 × phosphate buffer saline (PBS), and then immediately transferred and kept on ice for the determination of residual enzymatic activity (Xu et al, 2010).

### Measurement of antioxidant activity of recombinant protein

The SOD activity scavenging superoxide anions and hydroxyl free radicals was estimated using commercial kits (Nanjing Jiancheng Bio-Engineering Institute Co., Ltd.) following the manufacturer’s instructions. The 3-(4,5-dimethylthiazol-2-yl)-5-(3-carboxymethoxyphenyl)-2-(4-sulfonyl)-2H-tetrazolium/phenazine methosulfate (MTS/PMS) assay was used to measure the effects of HdhCu/Zn-SOD on L929 (a mouse fibroblast cell line) cell viability following a published study. L929 cells were cultured at 37 °C under 5% CO_2_ and then treated with different concentrations of the recombinant HdhCu/Zn-SOD protein (0, 12.5, 25, 50, and 100 µg/mL) for 24 h. The MTS/PTS cell proliferation working solution was added, and the cells were incubated in the dark for 0.75–2 h. The absorbance at 490 nm was measured using a microplate reader.

In the cell oxidative damage experiment, 150 µM H_2_O_2_ was used to treat L929 cells. Different concentrations of recombinant HdhCu/Zn-SOD protein (0, 12.5, 25, 50, and 100 µg/mL) were added, and nonhydrogen peroxide-treated cells were used as a blank control. The cells were incubated at 37 °C under 5% CO_2_ for 3 h before the MTS/PMS cell proliferation working solution was added. The aforementioned measurement method was used.

### Hemolysis experiment

The horse erythrocyte hemolysis experiment was carried out as previously described (Wu et al. 2019). A 4% horse erythrocyte suspension was prepared in PBS solution. Different concentrations of HdhCu/Zn-SOD (0, 7.5, 15, 30, 60, 120, 240, 480, and 960 μg/mL) were treated with an equal volume of horse erythrocytes at 37 °C for 2 h. PBS (0 μg/mL) and Triton X-100 were used as negative and positive controls, respectively. After centrifugation at 900 × *g* for 5 min, 200 μL of the supernatant of each concentration was transferred to a 96-well plate. The OD values were measured at 500 nm. Each concentration was prepared in quadruplicate. The percentage of hemolysis was calculated using the following equation: %hemolysis = (A—A0)/(AX—A0) × 100, where ‘A’ is OD at 500 nm with HdhCu/Zn-SOD solution, ‘A0′ is OD at 500 nm in PBS, and ‘AX’ is OD at 500 nm with 0.1% Triton X 100.

### Statistical analysis

One-way ANOVA and the independent-samples t-test were performed to compare the differences in relevant data using SPSS software (version 24.0). The results were considered as significant when p < 0.05. The asterisks indicate *p < 0.05, **p < 0.01, and ***p < 0.001. All data and graphs are expressed as the mean ± standard error.

## Results

### Tissue distribution of HdhCu/Zn-SOD mRNA

**Fig. 1:**
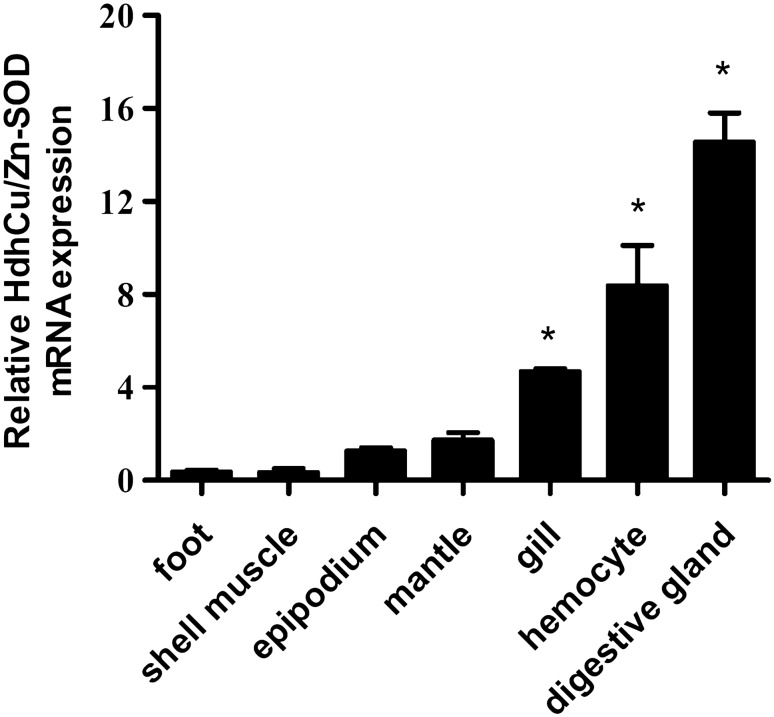
Expression of HdhCu/Zn-SOD in various Pacific abalone tissues and organs. β-actin was used as a housekeeping gene. The bars show the standard errors of mean values. These results were analyzed using an independent-samples *t*-test and asterisks represent significant differences compared to the shell muscle (*p < 0.05)

To investigate the transcript distribution of HdhCu/Zn-SOD among various tissues, qPCR was performed. The HdhCu/Zn-SOD mRNA exhibited a broad tissue distribution, being found in the hemocytes, gills, mantle, digestive glands, and shell muscle, etc. The HdhCu/Zn-SOD mRNA showed the highest expression level in the digestive glands, hemocytes, and gills (p < 0.05, compared to the shell muscle). Poor expression was observed in the shell muscle and foot (Fig. 1).

### Expression characteristics of HdhCu/Zn-SOD in Pacific abalone hemocytes after LPS stimulation

**Fig. 2:**
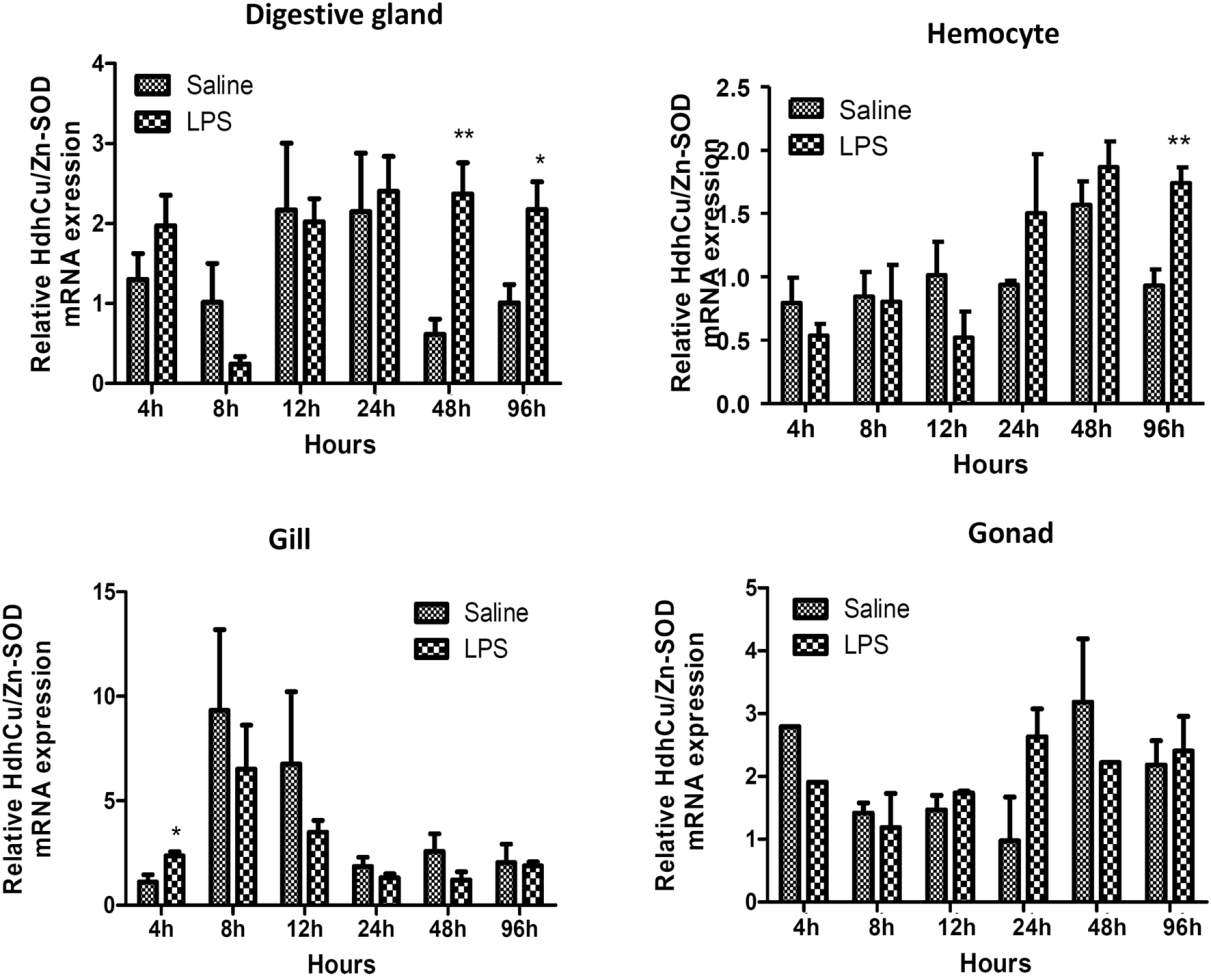
Time course of the mRNA expression of HdhCu/Zn-SOD in the hemocytes, gills, digestive glands, and gonads after LPS challenge. RPL7 was used as a housekeeping gene. The bars show the standard errors of mean values. These results were analyzed using an independent-samples *t*-test and asterisks represent significant difference compared with the saline group (*p < 0.05; **p < 0.01)

qPCR was performed to estimate the expression level of the HdhCu/Zn-SOD gene in response to LPS challenge. The HdhCu/Zn-SOD gene transcript was overexpressed in the digestive gland at 48 h (3.86-fold vs the control, p = 0.003) and maintained high expression until 96 h post-LPS injection (2.17-fold vs the control, p = 0.033) (Fig. 2). The mRNA expression pattern was different in hemocytes after LPS challenge, with upregulation observed at 96 h (1.87-fold vs the control, p = 0.003). The transcript level of HdhCu/Zn-SOD in the gills was upregulated at 4 h (2.13-fold vs the control, p = 0.026) and downregulated at other time points. These results confirm a tissue- and time-dependent expression pattern of HdhCu/Zn-SOD in abalones in response to LPS challenge.

### Construction and identification of the recombinant expression vector

**Fig. 3:**
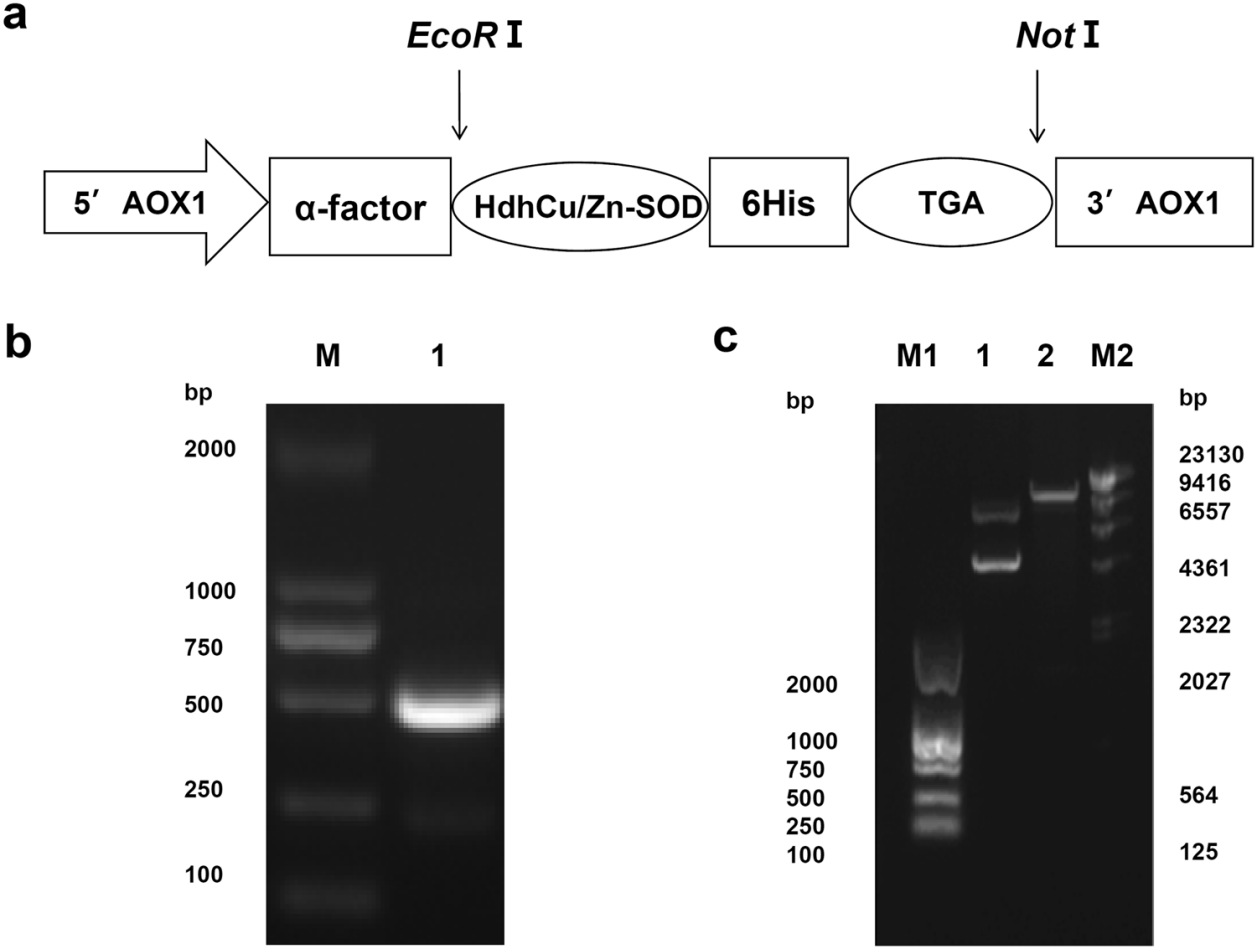
Construction of the recombinant expression vector. **a** Structural map of the PIC9K-HdhCu/Zn-SOD recombinant plasmid vector. **b** Electropherogram of the PCR product of the HdhCu/Zn-SOD expression fragment. Lane M: DL2000 Plus DNA Marker; lane 1: PCR product of HdhCu/Zn-SOD. **c** pPIC9K plasmid map after double enzymatic digestion. Lane M1: DL2000 Plus DNA Marker; lane 1: Before enzymatic cleavage of the vector; lane 2: After enzymatic cleavage of the vector; lane M2: λHindIII DNA Marker

The expression vector used in the experiment contains an AOX1 promoter and a yeast signal peptide α-factor that induces the secretion of the target gene. The six histidine residues that are fused at the C-terminus enable the target protein to be purified using 6 × His-Tag affinity chromatography. After the PCR-amplified HdhCu/Zn-SOD target gene was digested with EcoRI and NotI, it was ligated to the digested pPIC9K vector before transformation into *E. coli* DH5α. Positive clones were selected for sequencing by Sangon Biotech (Shanghai) Co., Ltd. The results showed that the ligation was correct, and the open reading frame (ORF) of the nucleotides was encoded continuously, which confirmed the amino acid sequence to be expressed. Figure 3a shows the construction of the pPIC9K-HdhCu/Zn-SOD expression vector.

The recombinant pMD18-T plasmid positive for HdhCu/Zn-SOD was used as a PCR template. The mature HdhCu/Zn-SOD peptide sequence (GenBank accession no. KX302627) was amplified by PCR, and a 462 bp PCR product encoding 154 amino acids was cloned into the pPIC9K expression vector (Fig. 3b). The amplified HdhCu/Zn-SOD target fragment was double digested with the restriction endonucleases *EcoR*I and *Not*I to obtain a gene fragment that could be ligated with pPIC9k containing the same sticky ends. The digested pPIC9K plasmid was loaded into a 0.8% (w/v) agarose gel and subjected to electrophoresis in Tris–acetate-EDTA buffer to measure the cleavage efficiency (Fig. 3c).

### Optimization of induction conditions for HdhCu/Zn-SOD expression

**Fig. 4:**
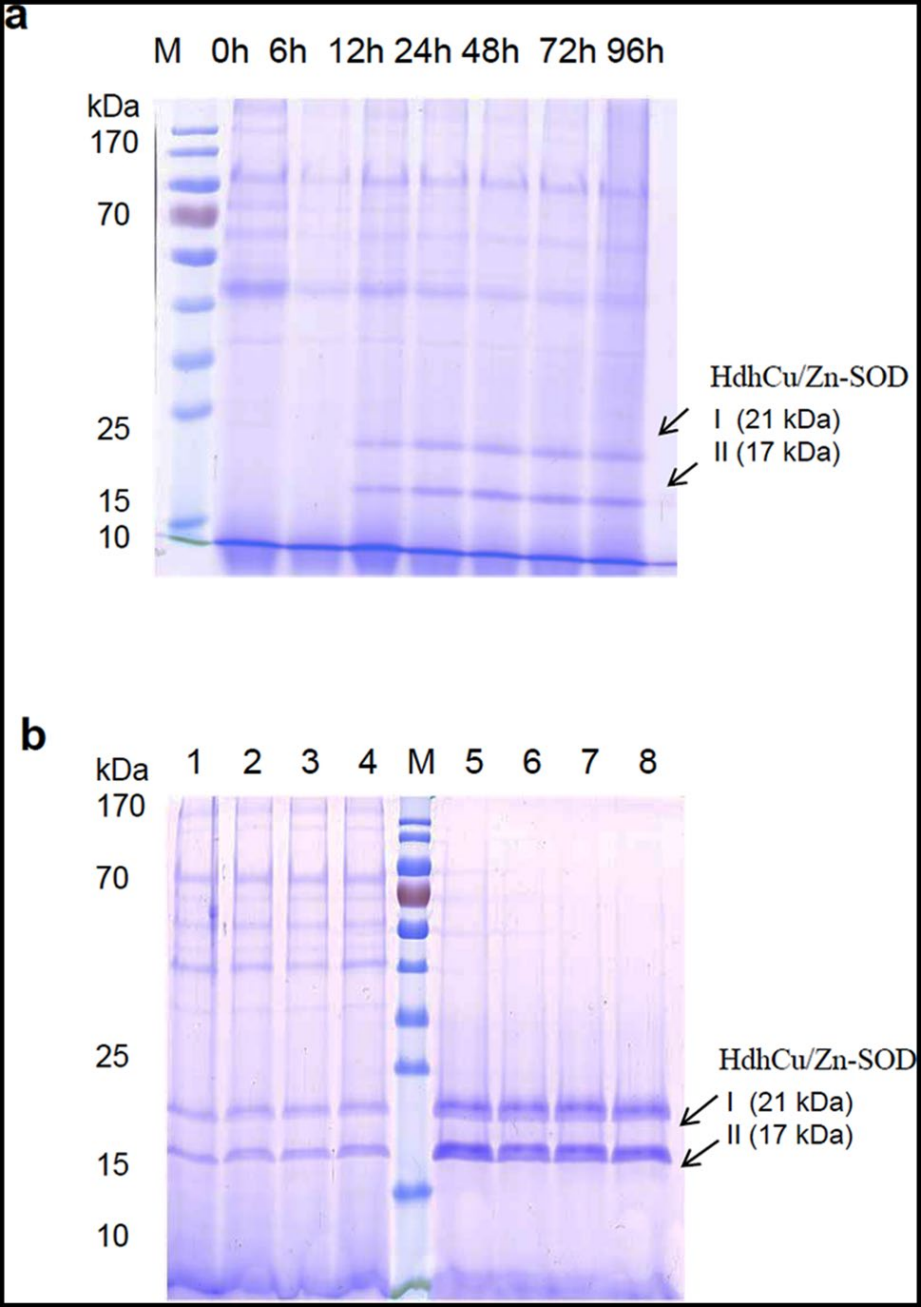
Recombinant expression and purification of the HdhCu/Zn-SOD protein. **a** The recombinant HdhCu/Zn-SOD expressed after different induction times (0 h, 6 h, 12 h, 24 h, 48 h, 72 h, and 96 h). **b** SDS-PAGE analysis of the purified HdhCu/Zn-SOD protein. Lane M: Protein marker 26,616 (Thermo Scientific); lane 1–4: Unpurified HdhCu/Zn-SOD protein.; lane 5–8: Purified HdhCu/Zn-SOD protein. Arrows show band I (21 kDa) and band II (17 kDa) of purified HdhCu/Zn-SOD protein

After the pPIC9K-HdhCu/Zn-SOD recombinant plasmid was transformed into *P. pastoris* GS115 cells, single clones were cultured in 10 mL of BMGY culture medium for 16 h until the logarithmic growth phase was reached, and the cells were collected by centrifugation. Next, we compared different expression durations (0 h, 6 h, 12 h, 24 h, 48 h, 72 h, or 96 h), pH levels of the expression medium (BMMY culture medium with pH 4, 5, 6, 7, or 8), and methanol concentrations at induction (0.5%, 1.0%, 2.0%, or 3.0%). The protein was expressed 12 h after methanol induction with a molecular weight of approximately 17 kDa and 21 kDa (Fig. 4a). In the range of methanol concentrations, the expression of the HdhCu/Zn-SOD protein was highest at 0.5% methanol (Supplementary Fig.S1a). The induction of HdhCu/Zn-SOD expression at different initial pH showed that the highest protein expression occurred at the initial pH of 6.0 (Supplementary Fig.S1b).

### Purification of the recombinant HdhCu/Zn-SOD protein

Immobilized metal-chelate affinity chromatography using nickel ions was employed for purification. HdhCu/Zn-SOD can specifically bind nickel ions in the affinity chromatography column. Based on the ultraviolet absorption curve detected at 280 nm, the wash components were impure proteins, while the elution component was the chelated target protein, HdhCu/Zn-SOD. The purified protein was subjected to SDS-PAGE electrophoresis (Fig. 4b), and the two target bands (approximately 17 kDa and 21 kDa) were excised for matrix-assisted laser desorption/ionization time-of-flight/time-of-flight mass spectrometry (MALDI-TOF/TOF–MS) protein mass spectrometry analysis (Supplementary Fig. S2). Comparison with the protein mass spectrometry database showed that the polypeptide fragments matched the amino acid sequence of HdhCu/Zn-SOD, demonstrating that the recovered bands were the target protein HdhCu/Zn-SOD.

### Assessment of HdhCu/Zn-SOD enzyme activity

**Fig. 5:**
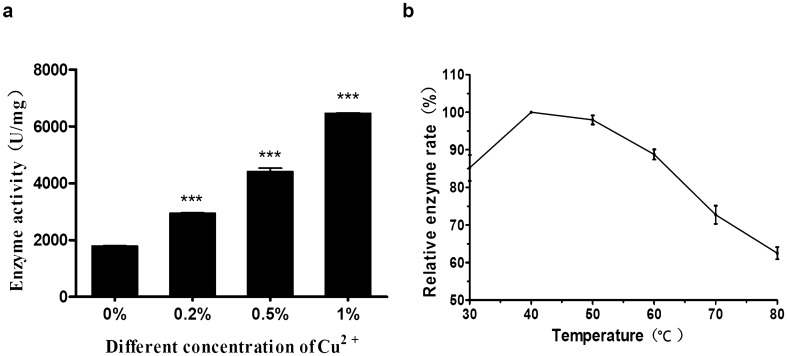
Effect of Cu ions and temperature on activity of HdhCu/Zn-SOD.** a** To determine the optimal ion concentration for recombinant expression, HdhCu/Zn-SOD enzyme activity under different Cu^2+^ concentrations (0%, 0.2%, 0.5%, and 1% CuSO_4_) was tested after culture for 24 h. **b** Effects of temperature on the activity of the recombinant HdhCu/Zn-SOD expression product were evaluated to determine the thermal stability of the enzyme. The recombinant expression product, HdhCu/Zn-SOD, was incubated at 30–80 °C for 10 min. These results were analyzed using an independent-samples *t*-test and asterisks represent significant differences compared to the 0% Cu^2+^ concentration (*p < 0.05,**p < 0.01, ***p < 0.001)

The HdhCu/Zn-SOD activities in the presence of Cu^2+^ were measured. The results showed that the enzymatic activity of HdhCu/Zn-SOD consistently increased with an increase in Cu^2+^ concentrations and showed statistical significance (p < 0.001) compared with the 0% CuSO_4_. HdhCu/Zn-SOD activity was the greatest at a 1% Cu^2+^ concentration (Fig. 5a).

The effect of temperature on SOD enzyme activity was measured. The maximum SOD activity measured was considered to be 100%. The SOD activity was above 80% at 30–65 °C. As the temperature gradually increased, the SOD activity of HdhCu/Zn-SOD decreased, reaching 62% at 80 °C. These results show that the HdhCu/Zn-SOD protein presents good thermal stability (Fig. 5b).

### The ability of recombinant HdhCu/Zn-SOD in scavenging superoxide anions and hydroxyl free radicals

**Fig. 6:**
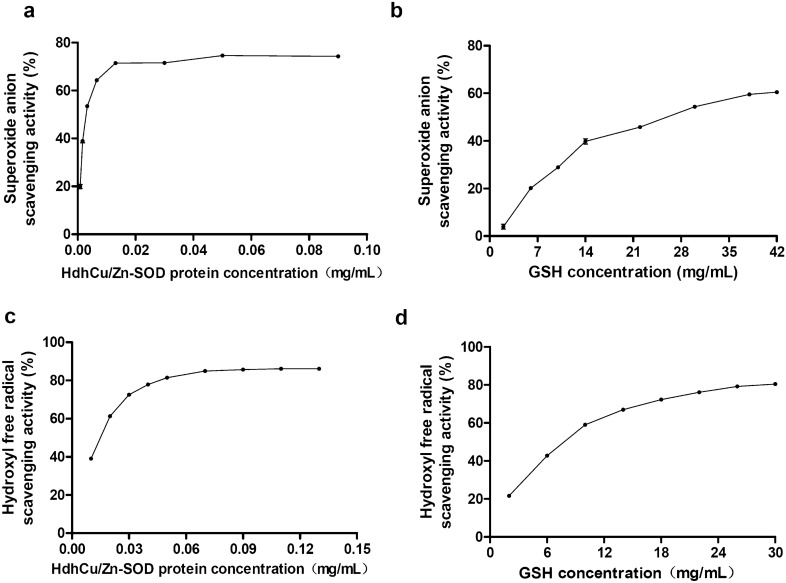
Comparison of quantitative ability of recombinant HdhCu/Zn-SOD and GSH to scavenge superoxide anions and hydroxyl free radicals. Superoxide anion scavenging ability of the recombinant HdhCu/Zn-SOD expression product **a** and GSH **b**. Hydroxyl free radical scavenging ability of the recombinant HdhCu/Zn-SOD expression product **c** and GSH **d**

As the HdhCu/Zn-SOD protein concentration increased, the superoxide anion scavenging activity of the HdhCu/Zn-SOD protein also increased. Dose-dependent scavenging is visible at up to 0.1 mg/mL, after which the scavenging becomes saturated (Fig. 6a). When the HdhCu/Zn-SOD protein concentration was 0.001 mg/mL, the superoxide anion scavenging activity was 50%. In comparison, the glutathione (GSH) concentration associated with the 50% superoxide anion scavenging activity was 30.35 mg/mL (Fig. 6b). The results showed that recombinant HdhCu/Zn-SOD presented a good superoxide anion scavenging ability.

The 50% hydroxyl free radical scavenging activity for the HdhCu/Zn-SOD protein was 0.016 mg/mL (Fig. 6c). When GSH was used as a positive control, the GSH concentration associated with a 50% hydroxyl free radical scavenging activity was 7.88 mg/mL (Fig. 6d). The results showed that the recombinant HdhCu/Zn-SOD protein possessed a strong hydroxyl free radical scavenging ability.

### Protective effects of recombinant HdhCu/Zn-SOD against H_2_O_2_-induced cellular damage

**Fig. 7:**
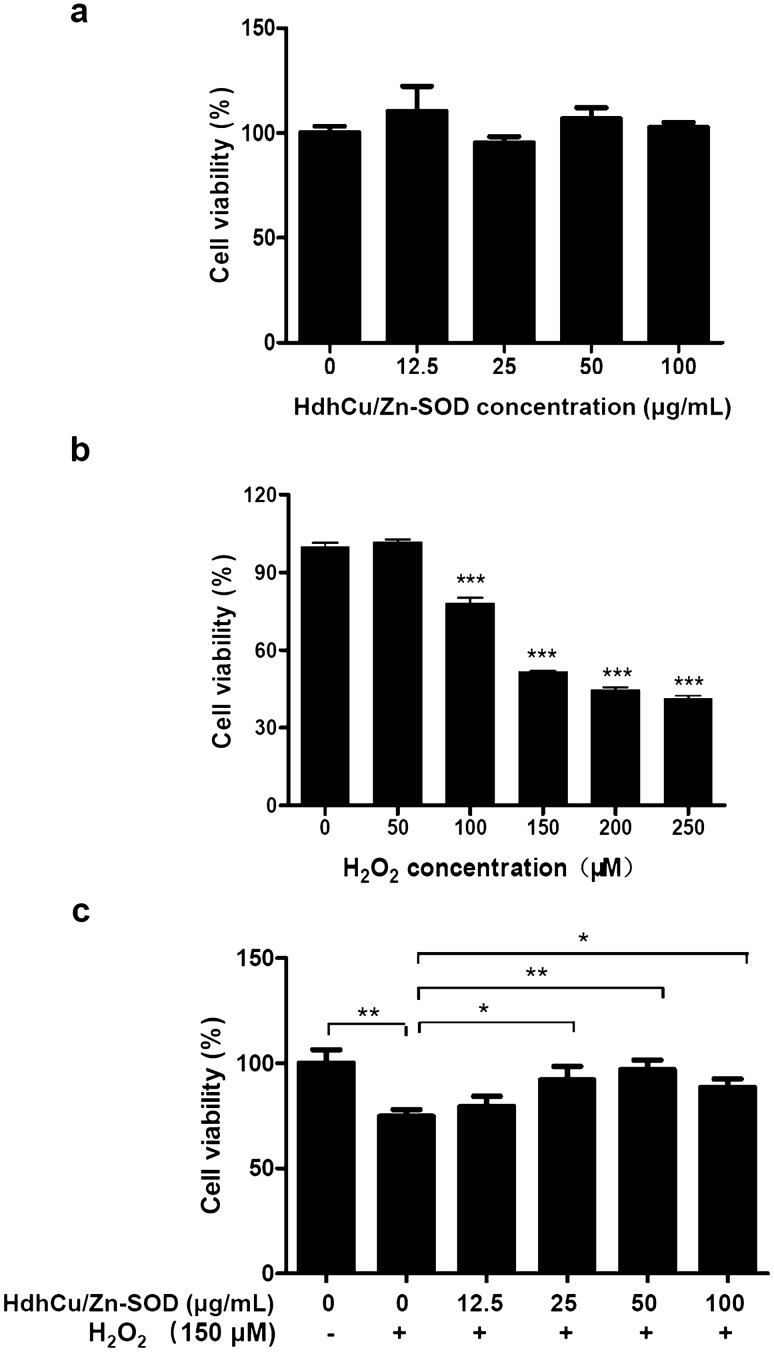
Effects of recombinant HdhCu/Zn-SOD on H_2_O_2_-induced cellular damage. **a** Treatment of L929 cells with recombinant HdhCu/Zn-SOD (0–100 µg/mL) showed no significant cell damage. **b** Treatment of L929 cells with H_2_O_2_ (0–250 µM). **c** Treatment of L929 cells with H_2_O_2_ (150 µM) dramatically decreased cell viability, whereas different concentrations of recombinant HdhCu/Zn-SOD (12.5–100 µg/mL) showed protective effects against H_2_O_2_-induced cellular damage. Cell viability was measured by the MTS/PMS assay. The results were analyzed using an independent-samples *t*-test and asterisks represent significant differences compared to the control (*p < 0.05, **p < 0.01, ***p < 0.001)

To determine whether the HdhCu/Zn-SOD protein reduces cell viability, different concentrations of purified HdhCu/Zn-SOD protein were added to L929 cells, followed by 24 h of culture. The MTS/PMS method was used to measure the effects of HdhCu/Zn-SOD on L929 cell viability (Fig. 7a). The results showed that HdhCu/Zn-SOD protein did not affect the viability of L929 cells at concentrations of 12.5–100 µg/mL relative to the normal cells (0 µg/mL, p > 0.05). L929 cells were treated with various concentrations of H_2_O_2_ (0–250 µM) for 3 h to determine the H_2_O_2_ concentration at which oxidative damage was induced. The cell viability was reduced in a concentration-dependent manner upon exposure to H_2_O_2_ (Fig. 7b). Normal cells (nonhydrogen peroxide (−) with no protein (0 μg/mL)-treated cells) were used as blank controls and the cell viability was considered as 100%. Treatment of L929 cells only with H_2_O_2_ (150 µM) significantly decreased cell viability (p = 0.008). Additionally, 150 µM H_2_O_2_ and HdhCu/Zn-SOD protein at varying concentrations were coincubated with L929 cells. As the HdhCu/Zn-SOD protein concentration (from 0 to 50 µg/mL) increased, the viability of the L929 cells continuously increased (Fig. 7c). The viability of L929 cells in the 25, 50, and 100 µg/mL HdhCu/Zn-SOD protein groups was higher than that in the 0 µg/mL HdhCu/Zn-SOD protein group. The 50 µg/mL HdhCu/Zn-SOD protein group showed 1.30-fold higher viability than the 0 µg/mL group (p = 0.005). Thus, HdhCu/Zn-SOD protein can protect L929 cells against peroxidation damage caused by H_2_O_2_.

### Hemolysis assessment of the recombinant HdhCu/Zn-SOD protein

**Fig. 8:**
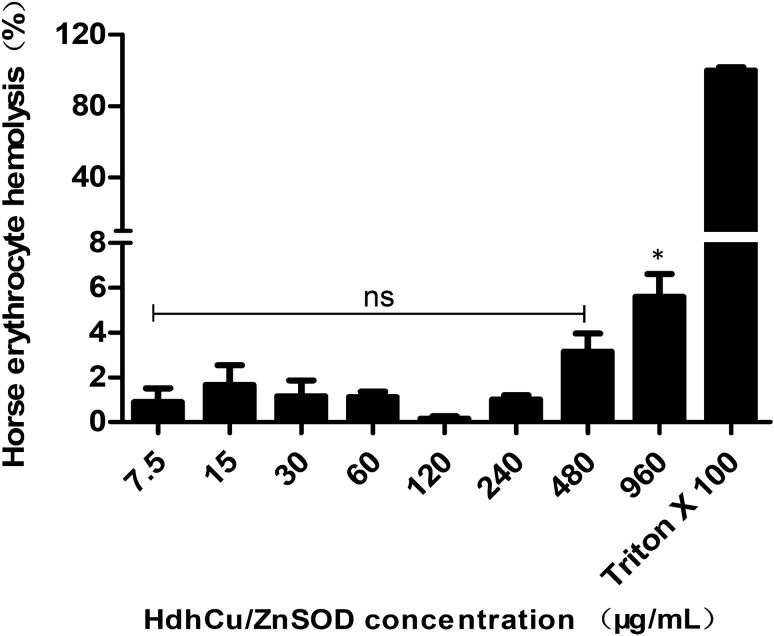
Hemolytic effects of the recombinant HdhCu/Zn-SOD expression product on horse erythrocytes

To evaluate the cytotoxicity of recombinant HdhCu/Zn-SOD protein, healthy mammalian red blood cells were treated with this protein. HdhCu/Zn-SOD was coincubated with horse erythrocytes for 2 h, followed by centrifugation. The supernatant was collected for OD_500_ measurement. The results showed that as the HdhCu/Zn-SOD protein concentration increased, no hemolysis occurred at protein concentration lower than 960 μg/mL (Fig. 8). Slight hemolysis was observed in horse erythrocytes at a concentration of 960 μg/mL, which is nearly 10 times the maximum working concentration (100 μg/mL).

## Discussion

The present study demonstrates that HdhCu/Zn-SOD is a ubiquitous enzyme, and the gene encoding this protein exhibits tissue-specific expression. We also found that HdhCu/Zn-SOD is up-regulated following LPS stimulation leading to oxidative stress. Furthermore, HdhCu/Zn-SOD activity is markedly increased in the presence of Cu^2+^ salts. In addition to this, HdhCu/Zn-SOD exhibits a good heat stability profile and is shown to have scavenging action against superoxide anion and hydroxyl radical in vitro.

Expression of the gene encoding HdhCu/Zn-SOD was detected by qPCR in all tissues of *H. discus hannai*, indicating that HdhCu/Zn-SOD may be a ubiquitous enzyme. We also found that HdhCu/Zn-SOD expression was the highest in the digestive gland, followed by hemocytes and gills, and was lower in muscle tissue. The digestive system of abalone consists of the digestive tract, and digestive gland (hepatopancreas) (Wigham 1976). As the main metabolic organ, the digestive gland is considered to be the major site for accumulation of toxic metals and bacteria, playing an important role in the regulation of oxidative stress and clearance of oxidative stress products. Hemocytes have been known as the important immune factor, playing an important role in cellular immunity in invertebrates, with the gill acting as a barrier to various exogenous stimuli. The digestive glands, gills, and blood are important immune organs; thus, the high expression of HdhCu/Zn-SOD in these tissues suggests that it may participate in the immune response against infection in mollusks. These results are somewhat similar to those reported by De Zoysa et al. (2009), who showed, using semi-quantitative RT-PCR, that *SOD of H. discus discus* was highly expressed in the digestive tract and gill. Similarly, for Mg-MnSOD and Mg-Cu/Zn-SOD of *Mytilus galloprovincialis* and Mm-icCu/Zn-SOD of *Meretrix meretrix*, transcripts were primarily detected in the hepatopancreas (Gao et al. 2012; Wang et al. 2013). The differences in tissue distribution profiles of Cu/ZnSOD expression may be attributed to the balance of various processes that act on the production of ROS in different environments.

LPS, a major component of gram-negative bacteria, induces proinflammatory transduction pathways, producing a strong immune stimulation response. To investigate the immunological function of HdhCu/ZnSOD in the abalone, we studied the transcriptional level of HdhCu/Zn-SOD after LPS stimulation. The results showed that HdhCu/Zn-SOD was up-regulated in the digestive gland, hemocytes, and gills, which confirmed the results of previous studies (Anju et al. 2013; Bao et al. 2009b Nikptiya et al. 2008). The phagocytic activity of an abalone infected with bacteria and foreign bodies produces a significant amount of ROS, which needs to be eliminated and this leads to additional transcription and translation of SOD (Nikptiya et al. 2008). Differences in SOD response to LPS among various tissues may be due to the diversity of isoenzymes.

In the present study, a recombinant HdhCu/Zn-SOD protein from *H. discus hannai* was successfully obtained in vitro by optimizing the expression conditions using a *P. pastoris* expression vector. After purification of the protein via immobilized metal affinity chromatography, two bands (approximately 17 and 21 kDa) were detected by SDS-PAGE and further confirmed by mass spectrometry. A similar observation has been documented in Sánchez-Venegas’s study, who found the *Deschampsia antarctica* recombinant SOD expressed by *P. pastoris* were showed two bands of 25 and 20 kDa. It was verified that SOD was secreted as a glycosylated protein by deglycosylation assays (Sánchez-Venegas et al, 2009). Given our results, the 17 kD protein corresponds to the estimated molecular weight for HdhCu/Zn-SOD and the 6xHis-tag. We speculate that the 21 kD band could be a glycoprotein of HdhCu/Zn-SOD.

Several research groups have employed prokaryotic expression systems (e.g. *E. coli*) to express recombinant SOD to determine its activity levels and investigate the role of SOD in marine mollusks. De Zosya et al. (2009) described overexpression of a recombinant Cu/Zn-SOD fusion protein in *E. coli* K12(TB1) cells, reporting an activity level of 2461 U/mg. In this study, we used a *P. pastoris* GS115 eukaryotic expression system for in vitro recombinant expression of HdhCu/Zn-SOD from the Pacific abalone. The enzymatic activity of purified HdhCu/Zn-SOD was greatly increased in the presence of Cu^2+^, reaching a maximum level of 6,771 U/mg in the presence of 1.0% Cu^2+^. These findings were consistent with those of a previous study by Xu et al. (2010), who reported that the expression level and activity of Cu/Zn-SOD from *Cristaria plicata* were increased by the supplementation of Cu^2+^ (or Zn^2+^) salts. These results showed that copper ions play an important role in improving SOD activity. Similarly, in *H. diversicolor*, the maximum activity of recombinant SOD was 801.8 U/mg protein in the presence of 1 μM Cu^2+^ and Zn^2+^ (Li et al. 2010). The activity of our recombinant enzyme was higher than that reported previously, possibly because of differences in recombinant expression vectors. *P. pastoris* is a good heterologous expression host that can more effectively modify proteins after translation compared to prokaryotic expression vectors (Ahmad et al. 2014; Macauley-Patrick et al. 2005).

We assessed the effect of temperature on SOD enzyme activity. HdhCu/Zn-SOD exhibited a good heat stability profile, with 80% of the optimal SOD activity maintained over a temperature range of 30–65 °C. The thermal stability of HdhCu/Zn-SOD was similar to that of other recombinant SOD enzymes, such as rCp-icCuZnSOD, a recombinant protein from a freshwater mussel, and rAi-icCuZnSOD, a bay scallop protein, which retained > 80% of the optimal activity below 60 °C (Bao et al. 2009b; Xu et al. 2010). As previously reported, our results confirm that SOD is a thermally stable enzyme.

Superoxide anions and hydroxyl radical, which can attack biological macromolecules and cause irreversible cell damage, are considered to be the main causes of oxidative damage (Zeng et al. 2018). As the first-line enzymatic defense against superoxide, SOD scavenges free radical species produced from endogenous and exogenous substances. To evaluate the antioxidant capacity of recombinant protein in vitro, we measured the ability of a recombinant HdhCu/Zn-SOD protein to scavenge superoxide anions and hydroxyl free radicals. By comparison with GSH (which is typically used as a scavenger positive control, Cai et al. 2015), we found that the purified HdhCu/Zn-SOD possessed a strong ability to scavenge superoxide anions and hydroxyl free radicals.

In this study, the cytotoxicity of the recombinant protein was evaluated, and it was found that HdhCu/ZnSOD did not change the activity of normal L929 cells. Furthermore, a cell peroxidation damage model of L929 cells was established. H_2_O_2_ is a stable ROS, which has previously been shown to inhibit the growth of L929 cells, resulting in their apoptosis (Ou et al. 2011; Shirato et al. 2016). Our results showed that HdhCu/Zn-SOD had a protective effect against hydrogen peroxide-induced cell damage, which may have been due to the ability of SOD to scavenge oxygen free radicals, whose production is stimulated by peroxide damage, in L929 cells, thus reducing the oxidative damage to cells. Similarly, Xu et al. (2010) carried out ethanol damage simulation experiments on cell models, which revealed that Cu/Zn-SOD from *Cristaria plicata* could protect L02 hepatocytes from ethanol damage.

An assessment of the recombinant HdhCu/ZnSOD protein showed that it did not cause hemolysis of horse erythrocytes, indicating that the enzyme was safe for cells. However, more comprehensive safety and efficacy evaluation is needed to future explore its application in cosmetic and medicine.

## Conclusion

The heterologous expression of HdhCu/Zn-SOD in *P. pastoris* and the antioxidant activity of this enzyme are reported for the first time. Recombinant HdhCu/Zn-SOD showed high enzyme activity and good antioxidant activity, as well as protective effects against H_2_O_2_-induced cellular damage. This protein is stable and safe at the cellular level, demonstrating its potential as a natural antioxidant agent for use in medicine and cosmetics.

## Electronic supplementary material

Below is the link to the electronic supplementary material.

Supplementary file1 Optimization of recombinant HdhCu/Zn-SOD expression in P. pastoris. a The effect of the methanol concentration (0.5%, 1%, 2%, 3%) on the expression of the recombinant HdhCu/Zn-SOD. b The effect of the initial pH (4, 5, 6, 7, 8) on the expression of the recombinant HdhCu/Zn-SOD. M: Protein marker 26616 (Thermo Scientific). Arrows show band I (21 kDa) and band II (17 kDa) of purified HdhCu/Zn-SOD protein. (TIF 4584 kb)

Supplementary file2 Mass spectrometry fingerprint of HdhCu/Zn-SOD band I (21 kDa) (a) and band II (17 kDa) (b). (TIF 32332 kb)

## Abbreviations

BMGY: Buffered glycerol-complex medium
BMMY: Buffered methanol-complex medium
cDNA: Complementary DNA
Cu/Zn-SOD: Copper and zinc SOD
Fe-SOD: Iron SOD
GSH: Glutathione
H_2_O_2_: Hydrogen peroxide
LPS: Lipopolysaccharide
MALDI-TOF/TOF–MS: Matrix assisted laser desorption/ionization time-of- flight/time-of- flight mass spectrometry
MD: Minimal dextrose
Mn-SOD: Manganese SOD
MTS/ PMS: 3-(4,5-Dimethylthiazol-2-yl)-5-(3-carboxymethoxyphenyl)-2-(4-sulfophenyl)-2H-tetrazolium/ phenazine methosulfate
Ni-SOD: Nickel-containing SOD
ORF: Open reading frame
PBS: Phosphate buffer saline
PCR: Polymerase chain reaction
ROS: Reactive oxygen species
SDS-PAGE: Sodium dodecyl sulphate polyacrylamide gel electrophoresis
SOD: Superoxide dismutase
YNB: Yeast nitrogen base
YPD: Yeast extract peptone dextrose medium
