# Supplementary figures and images for: Molecular characterization, purification, and antioxidant activity of recombinant superoxide dismutase from the Pacific abalone Haliotis discus hannai Ino
Source: World J Microbiol Biotechnol. 2020 Jul 14;36(8):115. doi: 10.1007/s11274-020-02892-5 (PMC7359182; doi:10.1007/s11274-020-02892-5)

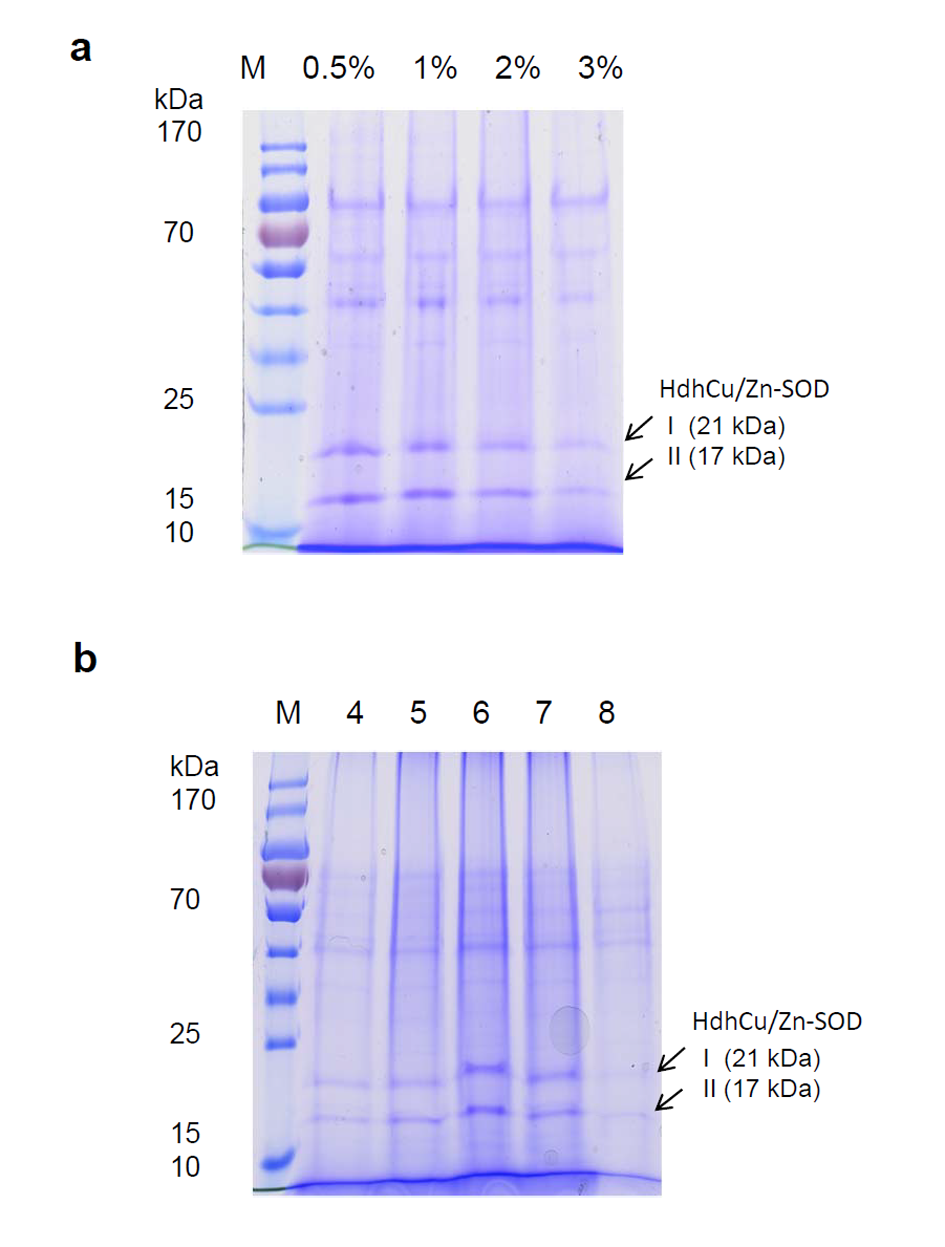

Supplement: Supplementary file 1 — Supplementary file1 Optimization of recombinant HdhCu/Zn-SOD expression in P. pastoris. a The effect of the methanol concentration (0.5%, 1%, 2%, 3%) on the expression of the recombinant HdhCu/Zn-SOD. b The effect of the initial pH (4, 5, 6, 7, 8) on the expression of the recombinant HdhCu/Zn-SOD. M: Protein marker 26616 (Thermo Scientific). Arrows show band I (21 kDa) and band II (17 kDa) of purified HdhCu/Zn-SOD protein. (TIF 4584 kb) [file 11274_2020_2892_MOESM1_ESM.tif]

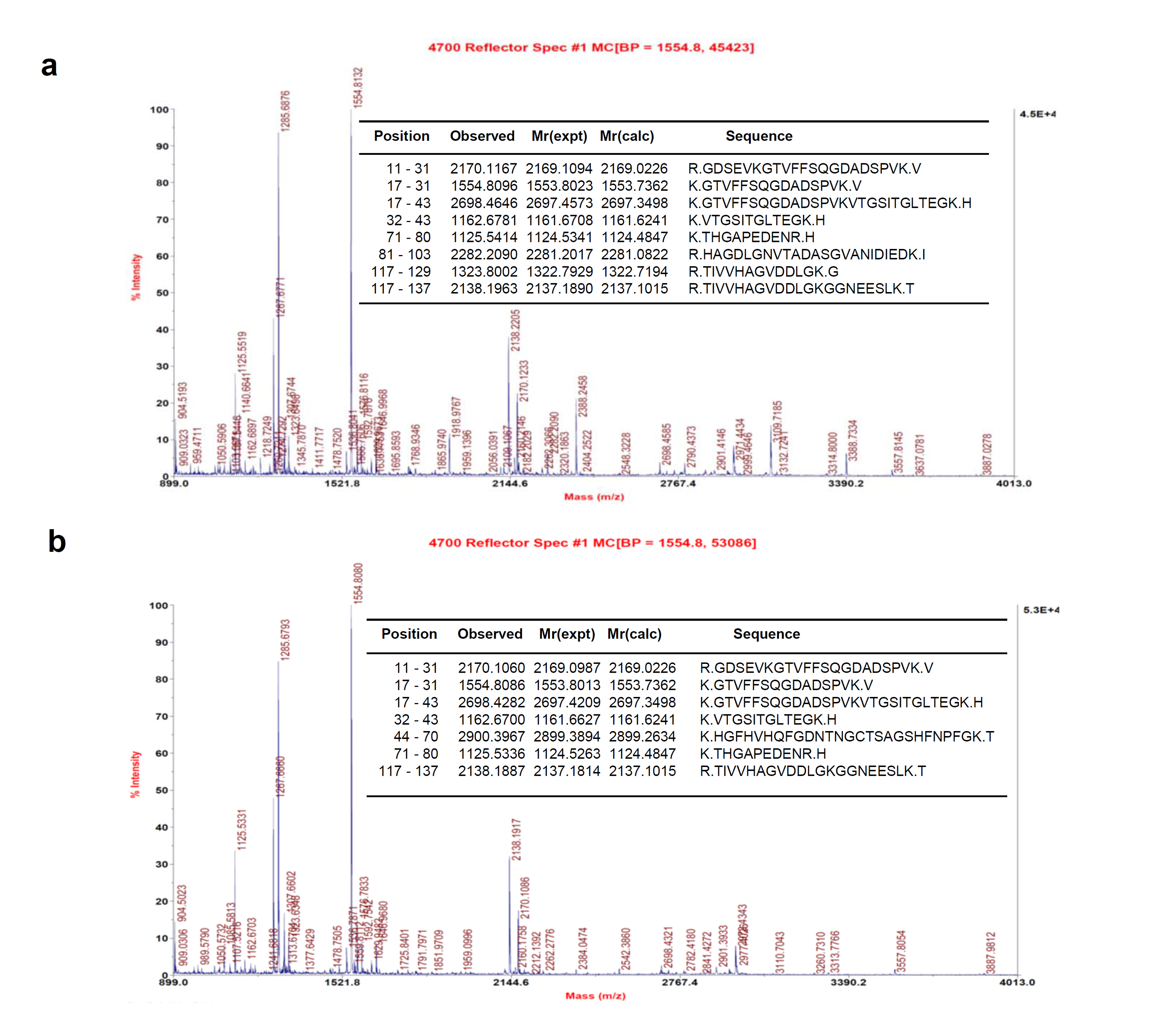

Supplement: Supplementary file 2 — Supplementary file2 Mass spectrometry fingerprint of HdhCu/Zn-SOD band I (21 kDa) (a) and band II (17 kDa) (b). (TIF 32332 kb) [file 11274_2020_2892_MOESM2_ESM.tif]
